# Left Inferior Frontal Gyrus Participates in Mediating the Renewal Effect Irrespective of Context Salience

**DOI:** 10.3389/fnbeh.2020.00043

**Published:** 2020-03-27

**Authors:** Silke Lissek, Anne Klass, Martin Tegenthoff

**Affiliations:** Department of Neurology, BG University Hospital Bergmannsheil, Ruhr-University Bochum, Bochum, Germany

**Keywords:** fMRI, extinction, renewal, context, inferior frontal gyrus, hippocampus, ventromedial PFC

## Abstract

The renewal effect of extinction demonstrates the context-dependency of extinction learning. It is defined as the recovery of an extinguished response occurring when the contexts of extinction and recall differ. Behavioral studies showed that modulating context relevance can strengthen context-specific responses. In our fMRI study, we investigated to what extent a modulation of context salience can alter renewal levels and provide additional information about the neural basis for renewal. In a within-subjects design, participants completed two sessions of an associative learning task in randomized order. In the salient condition (SAL), a context was presented alone at the start of each trial, before being presented together with the stimulus. The regular condition (REG) contained no context-alone phase. In about one-third of participants (SWITCH), the context salience modulation significantly increased renewal rates in the SAL compared to the REG condition. The other participants showed either renewal (REN) or no renewal (NoREN) in both conditions. The modulation did not significantly affect learning performance during the initial forming of associations or extinction learning. In the SWITCH group, activation in left opercular inferior frontal gyrus (iFG) during the recall phase was associated with a renewal effect, together with activity in the bilateral posterior hippocampus and ventromedial prefrontal cortex (vmPFC). Also during the extinction phase, left opercular iFG activation was higher in groups exhibiting renewal in recall, irrespective of the context salience modulation. Besides confirming the participation of vmPFC in extinction recall, our findings provide novel insights regarding an as yet undetected, potentially important role for renewal-supporting processes in left iFG during extinction learning and recall, which are presumably based on the region’s proposed function of evaluating competing response options under conditions of ambiguity.

## Introduction

The renewal effect of extinction is defined as the reoccurrence of an extinguished response when recall is performed in a context that differs from the one present during extinction learning (Bouton and Bolles, 1979). This context-dependency of renewal can pose serious problems for exposure therapies of phobias, since therapeutic success may not transfer from the therapeutic training setting to real-life situations, where previously extinguished phobic responses may reappear instead. Therefore, investigating internal and external conditions that modulate retrieval of extinction memory and evoke the renewal effect can support the development of effective therapies.

While most research on extinction learning and renewal has focussed on fear extinction, the renewal effect is also observed in non-fear related learning, such as instrumental and appetitive extinction in animals (Bouton and Peck, 1989; Bouton and Todd, 2014). In humans, renewal occurs also in cognitive tasks that require extinction and recall of acquired associations (e.g., Üngör and Lachnit, 2006, 2008; Lachnit et al., 2008). Importantly, in studies on extinction without a fear component, typically only a certain percentage (45–65%) of participants exhibit a renewal effect of extinction ( e.g., Lissek et al., 2013, 2016). This phenomenon makes non-fear related extinction tasks particularly useful for the investigation of renewal since opposing responses to an identical input can be analyzed. Firstly, the observation implies that participants showing and not showing renewal use different strategies in the processing of context (Lissek et al., 2016). In line with this, behavioral studies found that in the majority of participants, the propensity to exhibit or not to exhibit renewal remained stable over several sessions of the same task conducted at intervals of 1 to 4 weeks, thus suggesting a trait-like or enduring processing strategy. 87.23% of a sample of *n* = 47 healthy untreated participants showed an intra-individually stable response strategy over two sessions performed at an interval of 1 week: 63.83% showed renewal and 23.40% showed no renewal. Over four sessions (three at an interval of 1 week, the fourth after 4 weeks), 79.16% of a sample of *n* = 24 exhibited an intra-individually stable response strategy, with 70.83% showing renewal and 8.33% showing no renewal (Uengoer et al., in press). Also, contextual fear renewal showed high correlations across three sessions conducted at intervals of approximately 12 weeks (Zeidan et al., 2012), indicating that the stability of renewal behavior is not restricted to non-fear related tasks. Further supporting the assumption of an enduring, favored processing tendency is the finding that participants who show renewal consider the context not only during extinction, due to the unexpected change of contingencies that supposedly directs attention to the context (Bouton, 2004; Rosas and Callejas-Aguilera, 2006), but already during the acquisition of a context-related task, even though in this initial phase the context is not yet relevant (Lissek et al., 2016).

Neural correlates of renewal have been observed in brain regions processing context during extinction in both fear- and non-fear related learning, i.e., hippocampal and prefrontal areas. Context-dependent human fear extinction memory recruited hippocampus and ventromedial PFC for context encoding and retrieval (Kalisch et al., 2006; Milad et al., 2007). Besides its function for contextual encoding and retrieval of extinction memories (Bouton et al., 2006), the hippocampus also interacts with medial PFC to regulate context-specificity of extinction (Ji and Maren, 2007). Correspondingly, interindividual differences in the propensity to show renewal reflect in differential activation of the hippocampus and ventromedial PFC during a non-fear related predictive learning task: previous studies consistently found pronounced differences in hippocampal blood-oxygen level-dependent (BOLD) activation during acquisition and extinction between participants with and without a propensity for renewal (e.g., Lissek et al., 2013, 2016), linking higher hippocampal activity to subsequent renewal. Besides, these studies showed that ventromedial PFC was involved during extinction learning and extinction recall (Lissek et al., 2013, 2017) in the predictive learning task. Activity in ventromedial PFC during extinction learning in a novel context was positively correlated with renewal rates, suggesting that higher activation during encoding of these new associations entailed better assignment of the associations to their respective contexts during recall (Lissek et al., 2015a).

Another prefrontal region potentially important for renewal is inferior frontal gyrus (iFG)—an area which in our previous studies was occasionally found activated during extinction learning and recall (e.g., Lissek et al., 2017, 2019), and whose involvement in renewal is as yet unexplored. IFG is assumed to mediate response inhibition (Konishi et al., 1999), necessary during operant/instrumental extinction learning (Bouton et al., 2016). Both right- and left- hemispheric iFG have been implicated in processing response inhibition (Garavan et al., 1999; Swick et al., 2008; Hampshire et al., 2010). A broader role for left iFG is postulated by Novick et al. (2005) who suggest that it may be involved in general conflict resolution, specifically in detecting and resolving internal representational conflicts—making the region a likely candidate for processing conflicting response options during extinction and recall and thus involved in generating renewal.

Next to internal processing tendencies, also external characteristics of a task may influence the individual renewal propensity—such as the attributes of the context proper. The Attentional Theory of Context Processing (Rosas and Callejas-Aguilera, 2006) assumes that once attention is directed to a context, any information learned within this context should become context-specific. Thus, a crucial factor is the amount of attention dedicated to context stimuli, which again might be influenced by specific context characteristics. Correspondingly, attention to a context was found to modulate context-specificity of behavior (Uengoer et al., 2018). Relevant contexts received more attention (in terms of gaze duration), which led to more context-specific learning of humans in a behavioral predictive learning task (Lucke et al., 2013, 2014). Conceivably, a context attracting attention by its high visibility will appear more salient and also affect context-specific learning. Enhancing the visibility of the context by presenting it alone for 3 sec before each trial of the predictive learning task (Lissek et al., 2016) led to higher renewal rates than presenting the context and cue always together (Lissek et al., 2013). Moreover, enhanced hippocampal activation to the context-alone phase signaled that the context appeared more salient to all participants, regardless of their renewal tendency—corresponding to findings that found the hippocampus involved in processing context salience (Raza et al., 2017). Thus, while it cannot be excluded that presenting the context in this manner may evoke habituation instead of attention, in our study enhancing the visibility of the context arguably strengthened its perceived salience for participants.

While behavioral effects of context relevance upon context-related learning have been explored, imaging evidence of activity in extinction-relevant brain regions (other than hippocampus) to modulation of context salience is lacking. Moreover, previous studies used between-subjects designs which did not permit to identify, within the same individuals, potential differential effects of context salience variations upon neural correlates of extinction and renewal.

In this fMRI study, we, therefore, used a within-subjects design to investigate to what extent renewal behavior and associated BOLD activation in prefrontal and hippocampal regions can be modified by enhancing context salience. Healthy volunteers performed two sessions of a predictive learning task on two successive days: in one session, the context was presented always together with the stimulus [session regular (REG)]. In another session, with a different set of stimuli, the context was first presented alone, before the stimulus was added [session salience (SAL)]. The session order was randomized between subjects. Since renewal propensity was shown to be stable over repeated sessions, we predicted that the experimental modulation would cause behavioral and neural changes reflecting altered renewal processing and context salience appreciation. We hypothesized that the increase in context salience would: (a) strengthen the observed renewal effect—in terms of more participants exhibiting renewal and/or more renewal responses in the same individuals; and (b) increase the associated BOLD activation in hippocampal and prefrontal regions. Therefore, contrasting BOLD activation in renewal and no-renewal sessions of individuals whose renewal performance changed should highlight the regions crucially involved in processing renewal.

## Materials and Methods

### Participants

Fifty-one healthy volunteers without a history of neurological disorders (questionnaire, self-report) were recruited by local advertisements. Subjects were randomly assigned to the two groups performing different session sequences (REG–SAL or SAL–REG). After data acquisition, four subjects had to be excluded from further data analysis due to inadequate imaging datasets (bad signal or movement artifacts) or missing data. The reported analyses are calculated from the final sample of 47 participants (22 men, 25 women, mean age 25.12 years ± 4.368 st.dev., range 18–38 years). All participants had normal or corrected-to-normal vision and were right-handed [assessed using the Edinburgh Handedness Inventory (Oldfield, 1971). Participants received monetary compensation in the amount of 40€].

For data analyses, participants were identified as showing or not showing renewal separately for each session, based on their number of renewal responses (i.e., responding with the association correct during acquisition, see also “Behavioral Data Analysis” section) during the recall phase in trials designed to evoke renewal (i.e., ABA extinction trials). A participant who never, or in only a single response, showed renewal (i.e., who had 0%–10% renewal responses) was defined as a NoREN participant. This cutoff was chosen to account for a potential erroneous “renewal response” in a participant otherwise not tending to show renewal. A participant who showed a considerable percentage of renewal responses (40–100% renewal responses) was defined as a REN participant. Subsequently, based on the outcome of this procedure, each participant was assigned to one of the following groups: (a) showing renewal in both sessions (REN); (b) not showing renewal in any session (NoREN); or (c) showing renewal in only one of the sessions (SWITCH).

### Ethics Statement

All subjects participated in this study after giving written informed consent. The protocol was approved by the Ethics Committee of the Ruhr-University Bochum. The study conforms to the Code of Ethics of the World Medical Association (Declaration of Helsinki). Before the experiments, participants received handouts informing them about the fMRI procedure and had to complete a questionnaire checking their eligibility for MR scanning.

### Predictive Learning Task

**Figure 1:**
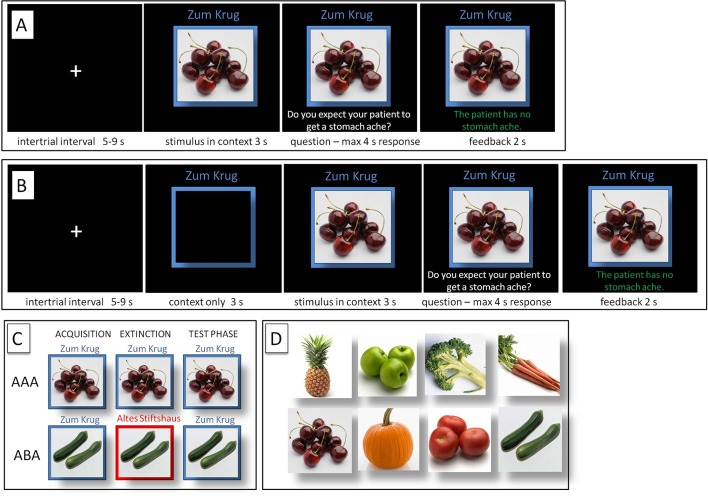
Predictive Learning Task. **(A)** Regular (REG) and **(B)** Salient (SAL) condition. **(C)** ABA and AAA conditions, with a context change in ABA. **(D)** Examples of food stimuli.

The predictive learning task (Üngör and Lachnit, 2006) used in this study is a task for context-related extinction learning without a fear component, suited to evoking a renewal effect by using an ABA design in the experimental condition, contrasted against an AAA design in the control condition. In the task, participants are asked to put themselves in the position of a physician and predict whether various food items served in different restaurants will lead to the aversive consequence of a stomach ache in their patient. Using this task, we closely followed the procedures of previous publications (e.g., Lissek et al., 2013, 2018) and thus are using similar text in the description. The task design is shown in Table 1 and Figure 1.

**Table 1:** Task design of the predictive learning task, with the conditions ABA and AAA and the learning phases acquisition, extinction, and test/recall (note that the classification of stimuli into extinction, retrieval, and new learning stimuli only applies from the extinction phase on).

		Acquisition		Extinction		Test/recall	
		Context 1	Context 2	Context 1	Context 2	Context 1	Context 2
AAA	Extinction	A+	B+	A−	B−	A?	B?
	Retrieval	C+	D−	C+	D−	C?	D?
	New learning	I−	J−	K+	L+		
		Q−	R+				
ABA	Extinction	E+	F+	F−	E−	E?	F?
	Retrieval	G+	H−	H−	G+	G?	H?
	New learning	M−	N−	P+	O+		
		S−	T+				

*“+” indicates the consequence of stomach ache, while “−” indicates no stomach ache. The “?” signals that no feedback is given*.

During the initial acquisition phase, participants learn to associate a presented food item with a consequence. In the regular (REG) version of the task (with context and cue presented always simultaneously), in each trial, a stimulus (photo of a vegetable or a fruit) is presented to the participant in one of two available contexts. The contexts consist of the restaurant names “Zum Krug” (The Mug, 1) and “Altes Stiftshaus” (The Dome, 2) and a frame in either red or blue color.

Sequence of a single trial: first, the stimulus in its context is presented for 3 s, then a question asking whether the patient will develop a stomach ache is superimposed on the frame, together with the response options “Yes” or “No”. Participants respond by pressing the respective button on an fMRI-ready keyboard (Lumitouch, Photon Control Inc., Richmond, BC, Canada) within a time window of 4 s. After the response, else after expiration of the response time, feedback with the correct answer is displayed for 2 s, i.e., “The patient has a stomach ache” or “The patient does not have a stomach ache.” The actual response of the participant is not commented upon.

The food stimuli are presented in randomized order. The acquisition phase contains 16 different stimuli, eight stimuli per context. Each stimulus is presented 8 times, amounting to a total of 128 trials. Half of the stimuli predict stomach ache, the others predict no stomach ache. The consequence of stomach ache is counterbalanced to appear equally often in both contexts.

During the extinction phase, half of the stimuli from the acquisition phase (8) are presented again. Of these, one half (4) is presented in the same context as during acquisition (condition AAA—no context change) and the other half (4) in a different context (condition ABA—context change) in randomized order. Within these groups of stimuli, a further distinction is being made between actual extinction stimuli (i.e., stimuli for which the consequence of stomach ache changes to no stomach ache during extinction) and retrieval stimuli (for which the consequence of stomach ache does not change), resulting in two extinction stimuli and two retrieval stimuli per context. Also, four new stimuli are introduced during the extinction phase, to balance the design so that it contains equal numbers of stimuli predicting stomach ache in both contexts. A further advantage of these new stimuli is that they allow investigating new learning in parallel to extinction learning. Overall, thus, the extinction phase contains a total of 12 different stimuli, six per context, i.e., two extinction stimuli, two retrieval stimuli and two new stimuli per context. Each stimulus is being presented eight times, amounting to a total of 96 trials. Again, half of the stimuli predict stomach ache, the others predict no stomach ache, and the consequence of stomach ache is counterbalanced to appear equally often in both contexts. In all other respects, trial design is identical to acquisition. Also, during all trial types in the extinction phase, participants receive feedback on the correctness of their response.

During the recall phase, extinction and retrieval stimuli are presented once again in the context of acquisition (five presentations per stimulus), resulting in a total of 40 trials. With the exception that during the recall phase participants receive no feedback with the correct response, trials are identical to those during acquisition.

For a detailed overview of the stimulus types, task phases, and context conditions, please refer to Table 1.

The version of the task with increased context salience (SAL) is identical to the regular version (REG), except for containing an additional phase at the beginning of each trial, during which the context is presented alone for 3 s. For the two sessions, two different stimuli sets were used to prevent memory interference effects.

### Procedure

In two fMRI sessions on two successive days, each participant performed two runs of the predictive learning task, one run in the REG condition, and one run in the SAL condition. To control for potential training or familiarity effects, the order of the conditions was randomized, so that half of the participants were tested in the order REG-SAL, the other half in the order SAL-REG.

### Imaging Data Acquisition

Functional and structural brain scans were acquired using a whole-body 3T scanner (Philips Achieva 3.0 T X-Series, Philips, The Netherlands) with a 32-channel SENSE head coil. BOLD contrast images were obtained with a dynamic T2* weighted gradient-echo EPI sequence using SENSE (TR 3,200 ms, TE 35 ms, flip angle 90°, field of view 224 mm, slice thickness 3.0 mm, voxel size 2.0 × 2.0 × 3.0 mm). We acquired 45 transaxial slices parallel to the anterior commissure-posterior commissure (AC-PC) line which covered the whole brain. High-resolution structural brain scans of each participant were acquired using an isotropic T1 TFE sequence (field of view 240 mm, slice thickness 1.0 mm, voxel size 1 × 1 × 1 mm) with 220 transversally oriented slices covering the whole brain. The task was presented to the participants *via* fMRI-ready LCD-goggles (Visuastim Digital, Resonance Technology Inc., Northridge, CA, USA) connected to a laptop that ran specific software programmed in Matlab (Mathworks, Natick, MA, USA). Responses were given through an fMRI-ready keyboard (Lumitouch response pad, Photon Control Inc., Richmond, BC, Canada).

### Imaging Data Analysis

For preprocessing and statistical analysis of fMRI data we used the software Statistical Parametric Mapping (SPM), Version 12 (Wellcome Department of Cognitive Neurology, London, UK), implemented in Matlab R2017b (Mathworks, Natick, MA, USA). Three dummy scans, during which the BOLD signal reached steady state, preceded the actual data acquisition of each session, thus preprocessing started with the first acquired volume. Preprocessing on single subject level consisted of the following steps: slice timing correction to account for time differences due to multislice image acquisition; realignment of all volumes to the first volume for motion correction; spatial normalization into standard stereotactic coordinates with 2 × 2 × 2 mm^3^ using an EPI template of the Montreal Neurological Institute (MNI) provided by SPM, smoothing with a 6 mm full-width half-maximum (FWHM) kernel, following the standard SPM procedure. The acceptable limit for head motion was 2 mm for translational movements and 0.5° for rotational movements.

In a first-level single-subject analysis we calculated activation during acquisition, extinction and recall phases, contrasted against baseline. We modeled regressors for the onset of each context-cue compound, question, and feedback during the REG and SAL condition, as well as the additional onset of the context alone during the SAL condition. All regressors were modeled using distinct stick functions convolved with the canonical hemodynamic response function in the general linear model implemented in SPM, in an event-related design. Contrasts used for the second-level analyses were based on the onset of the image of the context-cue compound and/or the context alone at the beginning of a trial, compared to baseline. The contrast images from the single-subject analyses were entered into a flexible factorial design containing the factors session (REG and SAL), renewal propensity (REN and NoREN) as well as different learning conditions for some analyses (e.g., context: identical/different; trial type: extinction, retrieval, new learning) for acquisition, extinction and recall phases. Also, we included the renewal rate as a covariate of interest in the design. We restricted our analyses to our *a priori* regions of interest, i.e., bilateral medial, ventral and orbital PFC, iFG and hippocampus. These regions were selected based on findings from previous studies (e.g., Kalisch et al., 2006; Lissek et al., 2013, 2018; Milad et al., 2007) in which they displayed significant participation in extinction and renewal, by processing context features, response selection/inhibition, and decision making. For these regions we constructed anatomical ROIs based on the corresponding anatomical regions defined in the WFU pickAtlas Toolbox implemented in SPM 12, using AAL atlas regions (Tzourio-Mazoyer et al., 2002). For data analysis, the respective ROIs of the anatomical regions were merged into a single contiguous ROI, which was used for small volume correction (SVC). The results are reported *p* < 0.05 FWE-corrected on peak level, minimum cluster size 20 voxel, with SVC.

### Behavioral Data Analysis

For all three learning phases, log files were recorded that contained information on response latency, response type and correctness of response, from which we calculated error rates during acquisition and extinction learning, overall rates as well as specific error rates for the different stimulus types (extinction, retrieval, and new learning stimuli). For calculation of the renewal effect, during the recall phase-only responses to stimuli with consequence change (extinction stimuli) were analyzed. The behavioral renewal effect in the predictive learning task is supposed to occur only in the condition ABA, due to the context change introduced during extinction learning. In case of renewal, associations learned during acquisition in context A will reappear in the recall phase which is again performed in context A, while extinction was performed in context B. In contrast, the AAA condition constitutes a control condition for extinction learning since here all learning phases are performed in an identical context. If extinction learning is successful, responses during the recall phase will reflect the associations learned during extinction. Only if extinction learning is impaired, responses in the AAA recall phase will reflect associations learned during acquisition. Errors in acquisition and extinction learning were defined as responses stating the incorrect association between the context-cue-compound and the consequence. During the recall phase, a response that referred to the association which was correct during acquisition constituted an error in the AAA condition and a renewal response in the ABA condition. Statistical analyses were performed using the IBM SPSS Statistics for Windows software package, version 23.0 (IBM Corp., Armonk, NY, USA). We performed ANOVA with repeated measures with the within-subjects factor condition (SAL and REG) for the acquisition and extinction phase to determine overall differences, paired *t*-tests to compare within-subjects performance in the groups, as well as two-sample *t*-tests to analyze between-groups differences. *χ*^2^ tests were used to determine proportions of men and women within the different groups. All results are quoted as mean ± SEM (standard error of means), unless stated otherwise.

## Results

### Behavioral Results

#### Participants Showing ABA Renewal Responses

For data analyses, participants were identified as showing or not showing renewal separately for each session, based on the presence of ABA renewal responses during the recall phase in trials designed to evoke renewal, i.e., ABA extinction trials (see also “Participants” and “Behavioral data Analysis” section for a detailed description). Each participant was then assigned to one of the following groups: (a) showing renewal in both sessions (REN); (b) not showing renewal in any session (NoREN); or (c) showing renewal in only one of the sessions (SWITCH).

In comparison of the conditions, we observed an increase in the proportion of participants showing renewal: from 25.53% in the REG condition to 53.19% in the SAL condition. Thus, of the participants showing no renewal in the REG condition, 37.14% changed their behavior to renewal in the SAL condition.

Overall, 27.65% (*n* = 13) showed renewal in only one session, i.e., they “switched” their mode of processing (SWITCH). The remaining participants were not affected by the manipulation of context salience: 25.53% (*n* = 12) exhibited renewal during both sessions (REN), while 46.81% (*n* = 22) showed no renewal during both sessions (NoREN).

There were no significant sex differences across the groups in renewal behavior ($\chi_{\left(1\right)}^{2}$ = 0.615, *p* = 0.735). Also within the groups, the proportions of men and women with and without renewal did not differ significantly: 50% of REN participants were women and 50% were men ($\chi_{\left(1\right)}^{2}$ = 0.000, *p* = 1.000). 59.1% of NoREN participants were women, 40.9% were men ($\chi_{\left(1\right)}^{2}$ = 0.727, *p* = 0.394). 46.15% of SWITCH participants were women and 53.86% were men ($\chi_{\left(1\right)}^{2}$ = 0.077, *p* = 0.782).

#### ABA Renewal Rates

An ANOVA with repeated measures showed for ABA renewal a significant main effect of group *F*_(2)_ = 105.420, *p* = 0.000, a main effect of condition *F*_(1)_ = 48.109, *p* = 0.000, and a significant interaction group*condition *F*_(2)_ = 44.933, *p* = 0.000.

**Figure 2:**
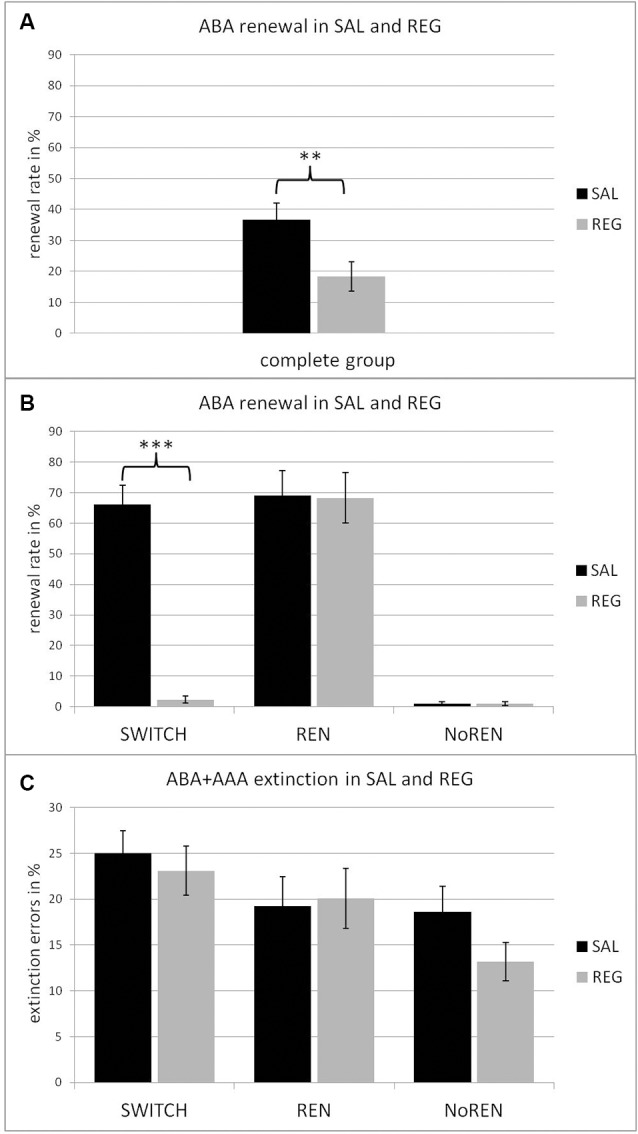
Renewal rates in the SAL (black) and REG (gray) conditions, in the complete group **(A)** and the three subgroups of SWITCH, REN and NoREN **(B)**. The data show that the significant difference in the complete group (*t*_(46)_ = 3.493, *p* = 0.001) is based on the change of renewal behavior in the SWITCH group, who exhibited significantly more renewal in the SAL than in the REG condition (*t*_(12)_ = 10.041, *p* = 0.000). **(C)** Percent of extinction errors in the SAL (black) and the REG (gray) condition in the three subgroups. An ANOVA with repeated measures revealed a significant main effect of group, a Bonferroni *post hoc* test demonstrated a significant performance difference between the SWITCH and the NoREN groups. **p < 0.01, ***p < 0.001.

Overall, the SAL condition evoked significantly higher ABA renewal rates than the REG condition, irrespective of session order (*t*_(24)_ = 3.970, *p* = 0.001, mean ABA renewal SAL 67.6% ± 4.98, mean ABA renewal REG 34.0% ± 7.81) in participants with a propensity for renewal (see Figure 2B). This effect was predominantly based on the performance of the SWITCH group, who reflected context salience in their renewal level (*t*_(12)_ = 10.041, *p* = 0.000; mean ABA renewal SAL 66.15% ± 6.36; mean ABA renewal REG 2.31% ± 1.22), while the REN group maintained the same level of renewal in both sessions (*t*_(11)_ = 0.088, *p* = 0.932, mean ABA renewal SAL: 69.16% ± 8.02, mean ABA renewal REG: 68.33% ± 8.33; see Figure 2B).

Still, the effects of context salience upon renewal were significant even when the complete group was considered (including NoREN subjects): the SAL condition evoked more renewal than the REG condition *t*_(46)_ = 3.493, *p* = 0.001 (mean ABA renewal SAL 36.66% ±5.46; mean ABA renewal REG 18.33% ±4.69; see Figure 2A).

#### AAA Errors in Recall

In the complete group, there was no significant main effect or interaction in AAA recall errors for extinction trials, according to an ANOVA with repeated measures for the SAL and REG sessions (main effect of condition: *F*_(1,44)_ = 1.023, *p* = 0.317; main effect of group: *F*_(2,44)_ = 1.601, *p* = 0.213; interaction condition*group: *F*_(2,44)_ = 0.920, *p* = 0.406).

Consequently, REN and SWITCH did not differ concerning their level of AAA errors, neither in the REG nor in the SAL session (REG: *t*_(23)_ = 0.214, *p* = 0.833; SAL: *t*_(23)_ = −0.680, *p* = 0.498).

#### Renewal Ratio

The renewal ratio describes the relation of responses during recall reflecting the association that was correct during acquisition, given in the ABA condition (i.e., renewal) compared to those given in the AAA condition. Since only ABA responses that reflect the association correct during acquisition indicate context processing, the renewal ratio is a measure for the context-dependency of recall responses. A ratio value of 1 indicates that every response of this type occurred during ABA recall and none during AAA recall—which suggests context consideration and thus genuine renewal. In contrast, a ratio value of −1 indicates that every response of this type occurred during AAA recall and none during ABA recall, suggesting rather weak memory for AAA extinction associations, with preserved memory for ABA extinction associations, but without context consideration. Higher ratios thus signal a higher probability of genuine renewal (for details on the calculation see Lissek et al., 2017).

The renewal ratio of the REN and SWITCH groups was similar in the SAL session where both groups showed ABA renewal (*t*_(23)_ = 0.356, *p* = 0.725; REN renewal ratio 0.783 ± 0.11 SEM; SWITCH renewal ratio 0.725 ± 0.12 SEM) in contrast to REG where the renewal ratios differed for obvious reasons (*t*_(23)_ = 5.646, *p* = 0.000; REN renewal ratio 0.843 ± 0.087, SWITCH renewal ratio −0.128 ± 0.143). Thus, both groups show a similar level of context-dependent responses in ABA renewal during the SAL session.

#### Learning Performance of Participants

Across the complete group, initial acquisition of associations as well as extinction learning were unaffected by the modulation of context salience: there were no significant differences in learning performance between SAL and REG sessions, irrespective of sequence (acquisition errors: *t*_(46)_ = 0.066, *p* = 0.948; extinction errors: *t*_(46)_ = 1.336, *p* = 0.188; retrieval errors *t*_(46)_ = 1.901, *p* = 0.064; new learning errors: *t*_(46)_ = 0.251, *p* = 0.803). Also within the subgroups REN, SWITCH, and NoREN there were no significant performance differences between the SAL and REG sessions. The results indicate that the salience of a context did not influence the learning process, neither during the initial forming of associations nor during the forming of new inhibitory associations during extinction learning (see Figure 2C and Table 2). While an ANOVA with repeated measures (conditions SAL and REG) did not find any significant differences in acquisition, for extinction learning the same analysis showed a significant main effect of group *F*_(2,44)_ = 4.293, *p* = 0.020, but no significant main effect of condition *F*_(1,44)_ = 0.954, *p* = 0.334 and no significant interaction *F*_(2,44)_ = 0.716, *p* = 0.494. A Bonferroni posthoc test revealed a significant difference between NoREN and SWITCH (*p* = 0.017) in extinction learning performance.

**Table 2:** Learning performance in acquisition and extinction—error rates in percent ± standard error of means.

	ACQUISITION		EXTINCTION		RETRIEVAL		NEW LEARNING	
	SAL	REG	SAL	REG	SAL	REG	SAL	REG
REN	16.86% ± 2.58	17.06% ± 2.52	19.27% ± 3.17	20.05% ± 3.27	10.68% ± 3.02	8.34% ± 1.65	9.64% ± 2.16	11.72% ± 1.77
SWITCH	19.71% ± 1.91	16.89% ± 1.64	25.0% ± 2.43	23.08%* ± 2.68	14.43% ± 3.80	10.82% ± 1.48	12.98% ± 2.91	12.26% ± 1.19
NoREN	13.74% ± 1.68	15.13% ± 1.95	18.61% ± 2.77	13.21%* ± 2.09	7.53% ± 1.45	5.12% ± 1.06	11.22% ± 1.72	9.80% ± 1.15
All participants	16.19% ± 1.19	16.11% ± 1.19	20.55% ± 1.69	17.69% ± 1.59	10.24% ± 1.49	7.52% ± 0.83	11.31% ± 1.25	10.97% ± 0.77
	**EXTINCTION ABA**		**EXTINCTION AAA**		**RETRIEVAL ABA**		**RETRIEVAL AAA**	
REN	16.67% ± 4.09	15.62% ± 3.48	21.87% ± 3.48	24.48% ± 4.17	12.50% ± 3.35	11.46% ± 2.41	8.85% ± 3.39	5.21% ± 2.64
SWITCH	28.36% ± 4.22	25.48% ± 2.87	21.63% ± 3.29	20.67% ± 3.27	15.38% ± 4.45	12.98% ± 2.06	12.98% ± 4.72	8.65% ± 1.94
NoREN	18.75% ± 3.24	13.64% ± 2.62	18.46% ± 3.27	12.78% ± 2.98	9.66% ± 2.00	4.83% ± 1.16	5.68% ± 1.74	5.40% ± 1.38
All participants	20.55% ± 1.69	17.69% ± 1.59	10.24% ± 1.49	7.52% ± 0.83	11.97% ± 1.76	8.78% ± 1.12	8.51% ± 1.77	6.25% ± 1.07

*Significant difference between NoREN and SWITCH.

#### Effects of the Repetition of the Learning Sessions

To determine whether the repeated performance of the task affected, we compared performance in the 1st and the 2nd session in an ANOVA with repeated measures with the within-subject factor session and the between-subject factor group:

For the acquisition phase, the ANOVA showed a main effect of session *F*_(1,44)_ = 13.430, *p* = 0.001, resulting from improved learning performance in the 2nd session compared to the 1st (*t*_(46)_ = 3.875, *p* = 0.000), presumably because of growing familiarity with the task. There was no significant main effect of group, and no significant session*group interaction. In extinction learning trials, the main effect of group *F*_(2,44)_ = 4.293, *p* = 0.020 pertained to the above mentioned significantly better performance of NoREN than SWITCH (Bonferroni *post hoc*, *p* = 0.017). There was no significant main effect of session and no interaction. In retrieval and new learning trials, there were no significant differences.

Concerning ABA renewal, an ANOVA with repeated measures revealed a significant main effect of session *F*_(1,44)_ = 10.856, *p* = 0.002, with overall higher ABA renewal rates in the 1st session than the 2nd (*t*_(46)_ = 2.707, *p* = 0.009), potentially since the novelty of the task attracted more attention to its features. In particular, ABA renewal rates were higher in the SAL session than in the REG session in the SAL-REG group (*t*_(22)_ = −4.830, *p* = 0.000), while in the REG-SAL group the ABA renewal rates in the SAL and REG sessions were not significantly different (*t*_(23)_ = −522, *p* = 0.607). This finding indicates that when the SAL condition was presented first, in a task that was new for the participants, it had more potential to bias processing towards a mode that supported renewal, presumably by attracting more attention. As expected, there was a main effect of group, due to the presence of the NoREN group who never showed renewal *F*_(2,44)_ = 105.420, *p* = 0.00, but no interaction between group and session.

### Imaging Results

To identify brain areas processing renewal-related information during recall and extinction learning, we focussed on activation differences in the hippocampus and prefrontal regions between the SAL and REG sessions by analyzing within- and between-subject differences in the recall and extinction phases. Besides, we calculated contrasts between the REN and the NoREN group for both sessions together, to identify renewal-related processing irrespective of context salience. The analyses reported for the extinction and recall phases include exclusively the extinction trials for the ABA and AAA conditions. The new learning and retrieval trials were not included in the analyses.

#### Recall Phase—Higher Activation in SAL Compared to REG Covarying With Renewal Level

This within-subjects contrast was calculated to identify regional activation that contributed to the shift from no renewal to renewal in the SWITCH group (see Figure 2) in the SAL condition compared to the REG condition. We compared BOLD activation that covaried with renewal level, elicited by the contrast SAL>REG in the SWITCH group, who showed renewal only in the SAL condition, and in parallel calculated the same contrast for the REN group. This second contrast served as a control to determine whether there would be similar activation differences between SAL and REG when participants showed renewal in both conditions.

**Figure 3:**
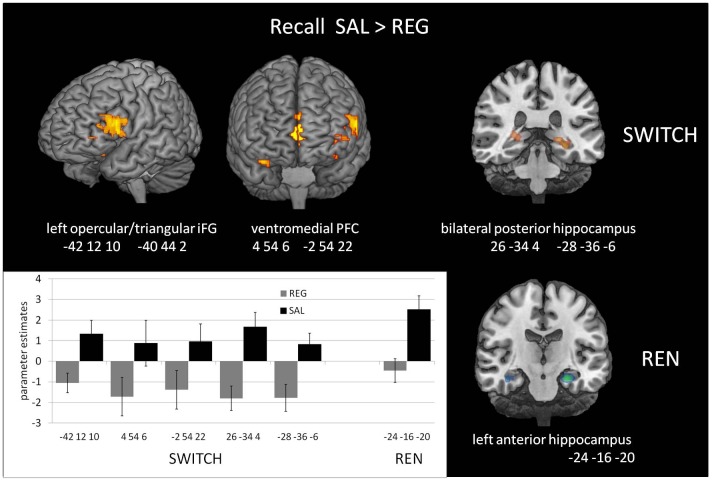
Increased Blood-oxygen level-dependent (BOLD) activation in the SAL session compared to the REG session during the recall phase, covarying with renewal level, in the SWITCH group (left), who showed renewal only in the SAL session, and the REN group (right), who showed renewal in both SAL and REG sessions. In the SWITCH group, left opercular/triangular inferior frontal gyrus (iFG) and ventromedial PFC as well as bilateral posterior hippocampus were recruited in the SAL session where they showed renewal, while these areas were deactivated during the REG session without renewal. In the REN group, the pronounced activation in left opercular iFG was missing, underlining its role for processing renewal. Here, only the left anterior hippocampus exhibited differential activation in the sessions.

The SWITCH group exhibited higher BOLD activation in the SAL session in the left iFG (opercular and triangular part) as well as in ventromedial PFC, compared to REG. In the REN group, a differentiation between SAL and REG in these regions was lacking, underlining that the higher activation displayed in the SWITCH group during the SAL session in left iFG and ventromedial PFC was renewal-related. In the bilateral posterior hippocampus, the SWITCH group’s activation was also higher in SAL than in REG, signaling processing related to context retrieval during recall. In contrast, REN displayed no differential activation in the posterior hippocampus, presumably since here a similar hippocampal contribution was required in both SAL and REG sessions in the processing of context information supporting renewal (see Table 3 and Figure 3). However, in the REN group, the left anterior hippocampus exhibited differential activation in SAL compared to REG.

**Table 3:** Areas of higher activation in the SAL condition relative to the REG condition during the recall phase covarying with renewal level for within-subject contrasts in the SWITCH and REN groups (paired *t*-tests, SVC FWE-corrected *p* < 0.05 on peak level).

RECALL SAL > REG	SWITCH						REN						
Brain Area		MNI coordinates			Voxel	*t*	*p*	MNI coordinates			Voxel	*t*	*p*
		*X*	*Y*	*Z*				*X*	*Y*	*Z*			
Inferior frontal gyrus (opercular part)	L	−42	12	10	366	4.65	0.022						
Inferior frontal gyrus (triangular part)	L	−40	44	2	52	4.56	0.031						
(ventro) Medial PFC superior frontal gyrus	L	−2	54	22	40	4.75	0.014						
Ventromedial PFC	R	4	54	6	248	5.20	0.002						
Hippocampus	L	−28	−36	−6	145	5.13	0.002	−24	−16	−20	151	5.66	0.000
	R	26	−34	4	52	4.51	0.038						

In the NoREN group, only the left anterior hippocampus exhibited differential activation in SAL compared to REG (−30 −16 −18, 129 voxels, *t* = 4.15, *p* = 0.014*). In the opposite contrast REG>SAL, no significant areas of higher activation in REG were found in the ROIs, neither in the complete group nor in the subgroups of SWITCH and NoREN and REN.

#### Recall Phase—Higher Activation in the Respective Renewal Group Covarying With Renewal Level

To further characterize renewal-related activation differences in the SWITCH group during the two recall sessions, we compared them with the respective other groups who showed the opposite renewal behavior in a given session, in the contrasts REN>SWITCH REG and SWITCH>NoREN in SAL.

When contrasting the SAL recall performance of the SWITCH group against the NoREN group, we again observed higher activation in left opercular/triangular iFG, together with left orbital iFG. The complementary contrast of the REG recall performance with the REN group (who showed renewal in the REG session while SWITCH did not), revealed significantly higher activation in the left posterior hippocampus, while activation in bilateral orbital iFG did not survive the FWE-corrected threshold. The only area activated commonly in both contrasts is the left posterior hippocampus. The data suggest that despite differential overt renewal behavior in the REG session, activation in the REN and SWITCH groups did not differ much, presumably due to the recruitment of largely similar processing networks. On the other hand, activation of SWITCH differed substantially from NoREN in the SAL session in left iFG regions, underlining their contribution to renewal-related processing (see Table 4).

**Table 4:** Areas of higher activation during recall in the group showing renewal compared to the group not showing renewal in the respective session (two-sample *t*-tests, SVC FWE-corrected *p* < 0.05 on peak level).

RECALL	REN > SWITCH REG						SWITCH > NoREN SAL						
Brain Area		MNI coordinates			Voxel	*t*	*p*	MNI coordinates			Voxel	*t*	*p*
		*X*	*Y*	*Z*				*X*	*Y*	*Z*			
Inferior frontal gyrus (triangular/opercular part)	L							−58	22	12	128	4.05	0.047
Inferior frontal gyrus (orbital part)	L							−36	26	−14	121	4.47	0.045
	R										
Inferior frontal gyrus (triangular part)	L							−40	44	2	132	4.43	0.025
Hippocampus	L	−26	−36	−6	94	4.45	0.047						
	R												

#### Extinction Phase—Higher Activation in the Salient Than the Regular Context Covarying With Renewal Level

The purpose of the within-subjects contrast SWITCH EXTINCTION SAL>REG was to explore whether the differences in renewal level in SAL compared to REG have precursors in differential activation in relevant brain regions already during extinction learning. Such differences may signal a form of processing conducive to later renewal. The second within-subjects contrast REN EXTINCTION SAL>REG served as a control to determine whether activation differences would also occur when participants show renewal in both conditions—which would indicate that similar activation differences in the SWITCH group were not exclusively related to renewal.

**Figure 4:**
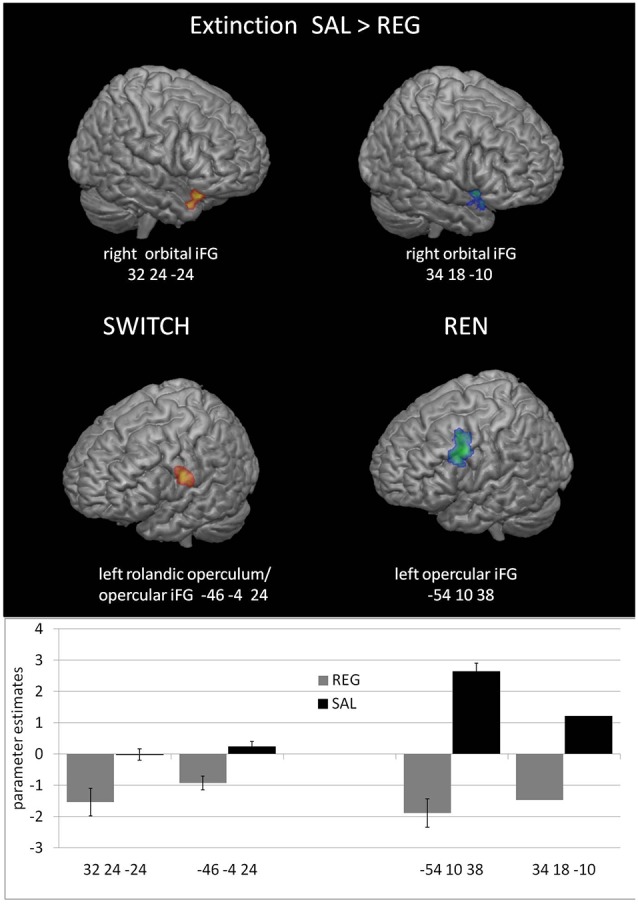
Increased BOLD activation covarying with the renewal level in prefrontal regions during the SAL session compared to the REG session in the SWITCH group (left) and the REN group (right).

Both the SWITCH and REN groups showed higher activation to SAL in right orbital iFG, and in a cluster including left opercular iFG/Rolandic operculum, suggesting that these regions already contribute to renewal-related processing during extinction learning. However, since the REN group exhibited renewal in both sessions, part of their increased iFG activation might be attributable to higher context salience. Significant hippocampal differences were not observed. In the REN group, this is presumably because the context was processed in both sessions—thus it is conceivable that also the SWITCH group processed the context in both conditions (see Table 5 and Figure 4). In the opposite contrast REG>SAL, no significant areas of higher activation in REG were found in the ROIs, neither in the complete group nor in the subgroups of SWITCH and NoREN and REN.

**Table 5:** BOLD activation in extinction trials covarying with renewal level, within-subjects contrast SAL>REG in the individual groups (paired *t*-tests, SVC FWE-corrected *p* < 0.05 on peak level).

EXTINCTION SAL > REG	SWITCH							REN					
Brain Area		MNI coordinates			Voxel	*t*	*p*	MNI coordinates			Voxel	*t*	*p*
		*X*	*Y*	*Z*				*X*	*Y*	*Z*	
Inferior frontal gyrus (orbital part)	R	32	24	−24	94	5.36	0.003	34	18	−10	360	4.46	0.037
Inferior frontal gyrus (opercular part)	L							−54	10	38	207	4.56	0.035
Rolandic operculum/inferior frontal	L	−46	−4	24	209	4.56	0.035						
gyrus (opercular part)													

#### Extinction Phase—Regions Processing Renewal Across Sessions, Unrelated to Context Salience

These contrasts served to identify regions that regardless of context salience exhibited differential activation in the REN and NoREN subgroups during extinction learning. Presumably, such between-subject differences should resemble those found in the within-subjects contrast of SWITCH in the comparison of the conditions in which they do and do not show renewal.

**Figure 5:**
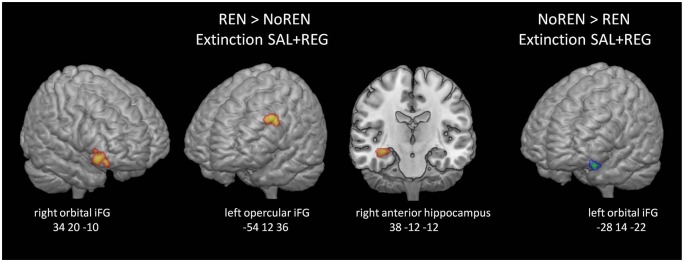
BOLD activation in extinction trials, comparison of REN and NoREN across the SAL and REG sessions. Higher activation in left opercular and right orbital iFG as well as the right anterior hippocampus in the REN group suggests renewal-related processing independent of context salience.

Activation differences unrelated to context salience between REN and NoREN participants were located in bilateral orbital iFG and a large cluster in left opercular iFG, as well as in the right anterior hippocampus. Here REN exhibited higher activity, suggesting that these regions were recruited by a processing mode that supported renewal. Only in a region in left orbital iFG, NoREN exhibited higher activation than REN across the sessions (see Table 6 and Figure 5).

**Table 6:** BOLD activation in extinction trials, comparison of NoREN and REN across the SAL and REG sessions (two-sample *t*-tests, SVC FWE-corrected *p* < 0.05 on peak level).

Extinction	REN > NoREN SAL + REG							NoREN > REN SAL + REG					
Brain Area		MNI coordinates			Voxel	*t*	*p*	MNI coordinates			Voxel	*t*	*p*
		*X*	*Y*	*Z*				*X*	*Y*	*Z*			
Inferior frontal gyrus (orbital part)	L							−28	14	−22	75	5.35	0.001
	R	34	20	−10	182	6.54	0.000						
Inferior frontal gyrus (opercular part)	L	−54	12	36	143	5.53	0.001						
Hippocampus	R	38	−12	−12	197	5.36	0.003						

#### Extinction Phase—Regions Processing Renewal-Supporting Information

To complement and support the within-subjects results shown in Table 3, this analysis aimed at determining between-subjects prefrontal and hippocampal regions that respond with more pronounced activation during extinction in a session that produced renewal. We compared the SWITCH group with the group showing the opposite renewal behavior in a given session: SWITCH was compared with REN in the REG condition (SWITCH no renewal, REN renewal) and again with NoREN in the SAL condition (SWITCH renewal, NoREN no renewal). Only in the contrast of REN and SWITCH in the REG session, regions in the right orbital iFG and right posterior hippocampus significantly differed in the activation.

While the REN>SWITCH result indicates that processing in iFG and hippocampus during extinction is relevant for later renewal, the lack of a difference in the SWITCH>NoREN contrast implies that BOLD activation differences between groups with and without renewal need not be very pronounced during extinction, which may imply that the differential processing leading to renewal occurs mainly during the recall phase (see Table 7).

**Table 7:** BOLD activation in extinction trials, contrasts REN>SWITCH REG and SWITCH>NoREN SAL to identify regions in the SWITCH group that show lower/higher activation in conditions with/without renewal (two-sample *t*-tests, SVC FWE-corrected *p* < 0.05 on peak level).

Extinction		REN > SWITCH REG						SWITCH > NoREN SAL					
Brain Area		MNI coordinates			Voxel	*t*	*p*	MNI coordinates			Voxel	*t*	*p*
		*X*	*Y*	*Z*				*X*	*Y*	*Z*			
Inferior frontal gyrus (orbital part)	R	34	24	−12	92	5.37	0.001						
Hippocampus	R	38	−28	−10	53	4.60	0.044						

## Discussion

### Enhancing Context Salience Induced More Participants to Show Renewal and Thus Increased Overall Renewal Rates

As hypothesized, the context salience manipulation evoked higher renewal rates across the complete sample in the SAL session compared to the REG session. This overall effect was based solely on the performance change in the SWITCH group, who showed renewal only in the SAL session, while the renewal rates in the REN group were not significantly altered by the context manipulation. The higher renewal rates observed in the SAL session complement behavioral studies (Lucke et al., 2013, 2014) by demonstrating that not only a relevant context, but also a particularly conspicuous context without specific informational value has the potential to strengthen context-specific learning. The behavioral results also resemble those of a previous study with a between-subjects design, in which the presentation of the context alone also yielded higher renewal rates (Lissek et al., 2016). Also, the SWITCH group results support accounts that posit an important role for attention to the context in the processing pipeline that leads to a renewal effect (Darby and Pearce, 1995; Rosas and Callejas-Aguilera, 2006; Uengoer and Lachnit, 2012).

The enhancement of context salience increased the proportion of participants showing renewal, from about 25% of the complete sample in the REG session to about 53% in the SAL session (by evoking renewal in 37% of the participants who did not show renewal in the REG session). Yet, even in the SAL session, 47% of the complete sample failed to show renewal. Thus, the findings also demonstrate that the mode of calling attention to context used in this study was not sufficient to influence all participants: while REN participants did not particularly benefit from the external attention boost to context, NoREN participants did not respond to it at all. Despite higher salience, the NoREN group may have considered the context as irrelevant, since it is not necessary for successful extinction learning. Else, instead of calling attention to it, the context presentation may have induced a habituation to the context in the NoREN group so they continued ignoring it. Further research is necessary to determine the reasons why NoREN participants did not respond to the context salience manipulation.

### Overview of Imaging Results

- The SWITCH group displayed higher activation in left (opercular) iFG during extinction learning and recall in the session with renewal than in the session without. In recall, this difference was absent in the REN group, who showed renewal in both sessions.
- Activation of left iFG during extinction learning also differed between groups showing/not showing renewal.
- The left iFG activation was largely independent of, i.e., not induced by, context salience, as comparisons of REN and NoREN across sessions showed.
- The SWITCH group’s higher activation of the hippocampus and vmPFC during recall in the session with renewal (i.e., the SAL condition) extends previous between-subjects findings that assigned an important role to these regions for renewal.

### Inferior Frontal Gyrus Activation During Extinction Learning and Recall Is Associated With Renewal

In the SWITCH group, left opercular iFG exhibited prominent activation that covaried with the renewal level during both extinction learning and recall in the SAL>REG contrast, as well as compared to the NoREN group during recall, and therefore was in all probability associated with a processing mode that promoted renewal. This assumption is supported by further contrasts who revealed: (a) no comparable difference in the REN group during recall, most likely because REN showed renewal in both sessions; and (b) higher left opercular iFG activation during extinction regardless of context salience: across sessions in the group showing renewal (REN>NoREN sal+reg), indicating that left iFG activation overall was largely independent of higher context salience. Yet, in the REN group, higher left iFG activation in the SAL>REG contrast during extinction learning hints at a minor influence of context salience at least in this learning phase.

Right orbital iFG, in the SWITCH group, was recruited more strongly during extinction learning in the SAL session compared to REG. The same differential activation was also found in the REN group, furthermore in the contrast of REN>NoREN across both sessions. In recall, no differential activation in this region was observed. Taken together, the findings suggest that right orbital iFG also participated in renewal-supporting processing predominantly during extinction. Activation in left orbital iFG was also observed when comparing NoREN with REN across extinction sessions, therefore suggesting no renewal-supporting processing. Besides, the lack of differential opercular iFG activation in REN>SWITCH REG during recall (i.e., the contrast analogous to SWITCH>NoREN SAL where this iFG activation was present), may indicate that the SWITCH group recruited this region also during REG recall, but a differential synergy of the processing network presumably attenuated its impact and thus prevented the generating of renewal.

The present results expand the findings from previous studies that too found—mainly incidental—iFG activation during extinction and recall (Lissek et al., 2015b; Klass et al., 2017; Lissek et al., 2019), by demonstrating for the first time differential activation of this region within the same individuals, depending on their momentary propensity to show renewal. This within-subject confirmation of previous between-subjects results highlights the important role iFG apparently has for processing renewal-related information.

The literature on proposed roles of iFG in human behavior describes functions of this region that are related to executive processing and may support renewal-related behavior. Right, iFG, in particular, has been implicated in response inhibition (Rubia et al., 2003; Aron et al., 2014), even though the notion of right iFG possessing a unique or specialized role for inhibition has been challenged recently (Hampshire et al., 2010; Hampshire, 2015). Accordingly, right iFG has been found to participate in more functions such as response selection between competing response options (Budhani et al., 2007; Mitchell et al., 2009), attentional control for responding to salient or task-relevant cues (Hampshire et al., 2010), as well as context monitoring (Chatham et al., 2012). Right orbital iFG is also engaged in learning stimulus-response rules (Toni et al., 2001; Aron et al., 2014). Also, left iFG appears to participate in response inhibition (Swick et al., 2008), conflict resolution (Novick et al., 2005) and decision processes (Arbula et al., 2017). In a comparison of left and right iFG functions, left iFG was commonly recruited when response selection was required in controlled responding, while right iFG was more involved when a task required response inhibition (Goghari and MacDonald, 2009). Others consider the observed functions as instances of an overarching role of iFG in modulation of stimulus-response maps by altering the weights of available response options to a stimulus, in order to facilitate optimal choice behavior (Greening et al., 2011). In contrast, the selection hypothesis considers left iFG as a general mechanism for selecting among competing representations (Thompson-Schill, 2003; Zhang et al., 2004). In any case, the role of bilateral iFG appears to be related to processing of response conflict and inhibition (Kemmotsu et al., 2005; van Veen and Carter, 2005) under attentional allocation.

In our study, higher activation in right iFG during extinction learning in participants with a propensity for renewal is consistent with its supposed functions of heightened attention towards salient stimuli, such as a changed context (Hampshire et al., 2010), of response inhibition (Goghari and MacDonald, 2009) under context monitoring (Chatham et al., 2012), and of response selection between competing response options (Budhani et al., 2007; Mitchell et al., 2009). Since REN participants always exhibited renewal, they presumably paid more attention to task-relevant cues and monitored the context—reflected in their higher right orbital iFG activation during SAL and REG sessions combined, compared to NoREN. Moreover, REN participants, as well as SWITCH participants in the session where they showed renewal, presumably realized that competing response options existed due to their encoding of context, while NoREN participants rather disregarded the previous response option once the new one was established, and therefore had no need of processing competing response options. The increased activity of right iFG during extinction learning in SWITCH and REN groups may also have reflected more pronounced response inhibition effects in the SAL condition.

In a study of source memory, activation of left opercular iFG was associated with the facilitation of further processing of previously encoded context information in situations where subjects succeeded in retrieving an item but were unsure about the context in which they encountered it before (Lundstrom et al., 2005). Following this explanatory approach, the SWITCH group’s higher recall activation in left opercular iFG during the SAL session, where they showed renewal, may have signaled ongoing retrieval of the contextual information previously associated with the stimulus in question, or perhaps also of the potential consequences associated with this stimulus. Such ongoing processing in left opercular iFG is compatible with the function of response selection from competing options, as proposed in the selection hypothesis of left iFG function (Thompson-Schill, 2003; Zhang et al., 2004).

Also in the source memory study (Lundstrom et al., 2005), a further region in left orbital iFG was considered—based on its activation pattern—as a kind of search engine for task-relevant information. In our study, left orbital iFG activation was not necessarily renewal-related, so a general function of searching task-relevant information is compatible with our findings.

In summary, activation of right orbital iFG during extinction learning may have been related to higher attention towards task-relevant cues and response inhibition. Prominent left opercular iFG activation during both extinction and recall in participants who showed renewal is consistent with a notion of this region being involved in selecting a response under conditions of perceived ambiguity, based on the processing of context information, and thus requiring response selection from competing options, a form of conflict resolution.

### Renewal Processing in Hippocampus and Prefrontal Regions

During recall in the SAL session, in which they showed renewal, compared to the REG session, in which they did not show renewal, the SWITCH group also displayed increased activation in ventromedial prefrontal cortex (vmPFC) and bilateral posterior hippocampus. During extinction learning and recall in the REG session, the SWITCH group’s activation in the posterior hippocampus was lower than the REN group’s.

Recall activation in vmPFC was observed previously in renewal participants, when compared with no-renewal participants (Lissek et al., 2013), reflecting predominantly retrieval of the association initially acquired in acquisition after extinction in a novel context compared to extinction in the identical context—i.e., renewal-related retrieval. Other studies found positive correlations between activation in the hippocampus and vmPFC during fear extinction recall (Milad et al., 2007), with activation in both the hippocampus and vmPFC being context-dependent (Kalisch et al., 2006). It has been assumed that vmPFC decides on the proper response based on contextual information it receives from the hippocampus (Corcoran and Quirk, 2007). Thus, the increased vmPFC activation found in the SWITCH group during SAL recall probably signaled the processing of context information to decide upon the association to be recalled, while during REG recall such a decision was not being made.

Pronounced context-related activation in hippocampus during extinction and recall is regularly found when comparing REN to NoREN groups in the predictive learning task (Lissek et al., 2013, 2016, 2018), consistent with studies on fear extinction and recognition tasks that found hippocampal activation necessary for the encoding of context information (Corcoran et al., 2005; Ji and Maren, 2007; Lambert et al., 2017). Bilateral posterior hippocampus activation is frequently observed associated with renewal (Lissek et al., 2013, 2016, 2018), and lacking activation or deactivation in this region may accompany reduced or lacking renewal (Lissek et al., 2017, 2018). Hippocampal activation in the SWITCH group in the present study is consistent with these findings: during extinction and recall in the REG session, where in contrast to the REN group they did not show renewal, their level of hippocampal activity was partially lower than in REN since context processing had a lower priority. Also within the SWITCH group, during recall bilateral posterior hippocampus was recruited more strongly in SAL than in REG, while during extinction no such within-group differences appeared. This result suggests that while the encoding of context may have occurred in both extinction sessions, hippocampal retrieval and procurement of context information was more pronounced during SAL recall, where it was required to support renewal. Thus, the potential of retrieving context information may have been available also in the REG session, but for some reason was not realized.

### Hippocampus, iFG, and vmPFC Acting in Concert During Recall

In all probability, the activation pattern of orbital and opercular iFG, hippocampus, and vmPFC observed in the SWITCH group in the SAL>REG contrast highlighted the regions whose cooperation is essential for generating renewal, by processing complementary task aspects during the extinction and recall phase.

(Functional) connectivity between these three regions in learning and recall has been observed in several studies, suggesting that cooperation between these regions mediates important aspects of encoding and recall. Functional connectivity between the posterior hippocampus and bilateral iFG was associated with the accuracy of memory performance and retrieval (Grady et al., 2003; Benetti et al., 2009; Manelis et al., 2013; Schedlbauer et al., 2014). Furthermore, effective connectivity between left hippocampus and left iFG was found to underlie generation and binding of semantic associations, with left iFG having a pivotal role in coordinating associative encoding processes (Addis and McAndrews, 2006). Thus, the interplay of the hippocampus and iFG during extinction presumably strengthened the forming of associations that included the context, and during recall improved retrieval of these associations. Furthermore, effective connectivity between the hippocampus and vmPFC controlled the choice of options from memory (Gluth et al., 2015), with the coupling between the hippocampus and vmPFC mediating a bias toward choosing better-memorized options. Such common activation of the hippocampus and vmPFC during recall was previously found not only in the predictive learning task but also in contextual fear extinction (Milad et al., 2007), suggesting cooperation of these regions in the retrieval of context- and cue-related information in line with the abovementioned findings. Resting-state studies found activity in vmPFC correlated with bilateral iFG (Uddin et al., 2009), and revealed functional connectivity between dorsal vmPFC and iFG (Jackson et al., 2020). Correspondingly, in the present study, the vmPFC clusters active during the SWITCH group’s SAL recall phase presumably communicated with both iFG and hippocampus in the processing of relevant task information.

In summary, the interaction between left iFG, vmPFC and hippocampus—regions which mediated context encoding and retrieval, evaluation of competing choice option, and control of option choice—presumably strengthened context-related association forming and retrieval based on context consideration, thus promoting the selection of an association that resulted in renewal.

### Conclusion

By comparing BOLD activation evoked by two versions of the predictive learning task with different levels of context salience, we identified brain regions whose collaboration appears essential to generating a renewal effect. Most importantly, increased BOLD activation in left (opercular) iFG during extinction learning and recall was associated with a switch from no renewal to renewal. This switch was presumably prompted by the region’s function of evaluating competing response options in a situation of perceived context-related ambiguity. The required context information was encoded and provided by the bilateral posterior hippocampus during extinction learning and subsequent recall, respectively. During recall, the vmPFC presumably integrated information delivered by iFG and hippocampus, promoting selection of the context-tied association that resulted in renewal.

The difference in left (opercular) iFG activation within individual participants or between groups, depending on whether they showed or did not show renewal, highlights an as yet unrecognized, essential role in generating renewal for functions mediated by this region, such as evaluation of and response selection from conflicting options.

Besides, our findings show that the renewal effect of extinction can be evoked by modulating context salience, indicating that taking the context into account for response selection is at least partly dependent on external factors.

In summary, the results from this study indicate that generating renewal depends on the interplay of the bilateral posterior hippocampus, ventromedial PFC, and—importantly—left iFG, which appears to evaluate competing response options and thus makes an essential contribution to the selection of a context-related response that results in renewal.

## Data Availability Statement

The datasets generated for this study are available on request to the corresponding author.

## Ethics Statement

The studies involving human participants were reviewed and approved by the Medical Ethics Committee of the Ruhr-University Bochum, registration no. 16-5738 dated 20.07.2016. The patients/participants provided their written informed consent to participate in this study.

## Author Contributions

SL: conceptualization, methodology, formal analyses, writing—original draft, writing—review and editing, and funding acquisition. AK: investigation, project supervision, and formal analyses. MT: conceptualization, resources, supervision, writing—review and editing, and funding acquisition.

## Conflict of Interest

The authors declare that the research was conducted in the absence of any commercial or financial relationships that could be construed as a potential conflict of interest.
